# Neural Tube Defects – *From Origin to Treatment*

**DOI:** 10.1186/1743-8454-3-6

**Published:** 2006-04-03

**Authors:** Carys M Bannister

**Affiliations:** 1 Higher Carr Farm, Hall Carr Road, Rawtenstall, Rossendale, Lancs, BB4 6BS, UK

## 

The title of this book shouts loud lofty aims. Does it fulfil them? It most certainly does, and in many ways it exceeds them. To whom is it of interest? Virtually everyone interested in spina bifida, be the reader a research worker, clinician, epidemiologist or anyone among a host of others concerned with spina bifida. By any standards it is a daunting task to cover one condition from its origin to treatment as the subtitle announces. The editor has chosen his contributors wisely, picking leaders in their fields that inevitably have given an account of their subject from a personal point of view. Most have also included for discussion, the apposing and sometimes the controversial views of others.

The book is divided into three sections: Basic Principles, Treatment and Education, and Public Health Issues.

The first section, Basic Principles, is further subdivided into four subsections, the first of which contains a group of papers, which are essential reading for anyone who wants or needs to update their knowledge of the embryology of neural tube development and the underlying biomolecular mechanisms. This is not easy reading but essential if one is to understand how and why things go wrong when spina bifida occurs. The second subsection deals with a variety of clinical features of neural tube defects. This subsection also includes papers concerned with the classification of the various manifestations of neural tube defects. Of particular interest are the myriads of syndromes that include spina bifida amongst their abnormalities. Do these conditions give a clue to the genetic defects in cases where spina bifida is the only abnormality? That is a hope not yet realised. Papers in the third subsection dedicated to the epidemiology of neural tube defects give a realistic view of the difficulties of collecting meaningful data and the strength and weaknesses of statistics based on these data. It is no longer a mystery why there is no consensus of opinion about whether the conception rate of spina bifida is increasing, staying the same or decreasing or indeed why the reported incidence of spina bifida in any particular country varies from one author to another. The last subsection deals with the genetics of neural tube defects. These chapters also do not make for easy reading for those not conversant with the names of the multitude of genes involved, but as future developments of the understanding, treatment and management of spina bifida are likely to come in this field of study, it is essential that all make themselves familiar with this work.

The second section is concerned with the Treatment and Education of patients with spina bifida. In this section the whole life cycle of patients is covered from the fetus to the all too often ignored adult. The first paper is a discussion of fetal surgery for myelomeningocele. Is this the way forward for the treatment of spina bifida? The answer to that question is still on hold, for although the numbers of fetuses treated is now several hundred, many aspects of the outcome are yet to be assessed and await the results of the randomised, controlled Management of Myelomeningocele Study (MOMS). It is gratifying that several of the papers in this section are devoted to the older patient with spina bifida and the associated problems of growing older, with the attendant difficulties of learning, separation from their parents and carers, and developing a degree of independence. There are no holds barred when it comes to discussing in detail the multiplicity of difficulties encountered by young persons with spina bifida. These range from deficits in brain function that cause learning difficulties which are carried over into the work place making it hard for them to successfully perform relatively simple jobs, to the social stigma of being incontinent of urine and faeces and having sexuality problems that affect interaction with peer groups. In this section there are two subjects that are surprisingly not included. The first is hydrocephalus, it is discussed in passing but one might have expected to have a chapter devoted to its management. The other area not mentioned is the management of the patient with severe brain malfunction. Their care as they grow older is no less important and worthy of consideration than that of less severely affected patients.

The third and last section in the book deals with the role of folic acid in the prevention of spina bifida and two other topics that are not unrelated to the former, namely the costs involved in the care of spina bifida patients and ethical issues raised by the condition.

Everyone, no matter what their expertise is in the field, have something to learn from what is written between the covers of this remarkable book. Don't take my word for it, go and get a copy and find out for yourself.

## Competing interests

The author(s) declare that they have no competing interests.

## Authors' contributions

Sole author

